# Sensory Adaptation and Short Term Plasticity as Bayesian Correction for a Changing Brain

**DOI:** 10.1371/journal.pone.0012436

**Published:** 2010-08-26

**Authors:** Ian H. Stevenson, Beau Cronin, Mriganka Sur, Konrad P. Kording

**Affiliations:** 1 Department of Physiology, Rehabilitation Institute of Chicago, Northwestern University, Chicago, Illinois, United States of America; 2 Department of Brain and Cognitive Sciences, Picower Institute for Learning and Memory, Massachusetts Institute of Technology, Cambridge, Massachusetts, United States of America; University College London, United Kingdom

## Abstract

Neurons in the sensory system exhibit changes in excitability that unfold over many time scales. These fluctuations produce noise and could potentially lead to perceptual errors. However, to prevent such errors, postsynaptic neurons and synapses can adapt and counteract changes in the excitability of presynaptic neurons. Here we ask how neurons could optimally adapt to minimize the influence of changing presynaptic neural properties on their outputs. The resulting model, based on Bayesian inference, explains a range of physiological results from experiments which have measured the overall properties and detailed time-course of sensory tuning curve adaptation in the early visual cortex. We show how several experimentally measured short term plasticity phenomena can be understood as near-optimal solutions to this adaptation problem. This framework provides a link between high level computational problems, the properties of cortical neurons, and synaptic physiology.

## Introduction

The excitabilities of individual neurons fluctuate over timescales ranging from milliseconds to hours due to effects such as electrical stimulation [c.f. 1], neuromodulator concentrations [2] , oxygen concentration [3] and circadian rhythms [4]. Such fluctuations are widely observed in experimental recordings where spike trains are collected over many trials in response to the same repeated stimulus [c.f. 5]. If a neuron encodes features of stimuli in the external world, such as the orientation of edges in the visual scene, then such fluctuations are a major computational problem. The firing rate of the neuron will depend not only on the properties of the stimulus but also on the fluctuations in the excitability of presynaptic neurons. Since such dependence would introduce noise and bias into neural activities we would expect the nervous system to correct for these fluctuations.

Here we consider adaptation as an estimation problem where neurons attempt to produce stable responses in the presence intrinsic fluctuations. That is, neurons must distinguish changes in sensory stimuli from fluctuations in the excitability of presynaptic neurons. This distinction may be possible if excitabilities and sensory stimuli change over time in different ways. If these two sources of fluctuations in presynaptic activity can be distinguished the noise introduced by excitability fluctuations can be removed. Given observations of presynaptic activity, we first consider the statistical problem of estimating presynaptic excitability. We then assume that, in order to reliably represent sensory drive, the postsynaptic response is the presynaptic activity normalized by the estimated excitability. In many ways this model provides an instantiation of normalization theories of adaptation where the nervous system attempts to correct low-level abnormal responses [6]. In the framework we propose here, adaptation is the result of a strategy to compute reliably by a nervous system that changes on many timescales.

Using this excitability estimation framework we examine a range of physiological adaptation phenomena. We examine short-term synaptic depression at a single synapse and medium-term tuning curve adaptation in early visual cortex. Experimental results in both these domains are well-described by a model that implements excitability estimation at the level of single synapses.

Recently, neuronal adaptation has been treated as a mechanism that allows the nervous system to accurately represent stimuli in the face of changes in the statistics of the external world [7], [8]. At a high-level, this approach would allow the nervous system to reduce redundancy [9] and maximize the amount of sensory information transmitted [10]. Rather than examining adaptation to extrinsic changes, here we ask which adaptation rules would optimally remove the effects of intrinsic fluctuations in pre-synaptic excitability. We find that adaptation to intrinsic fluctuations can reproduce experimental observations of a number of adaptation phenomena. This raises the important possibility that adaptation may be not only a mechanism for matching the statistics of the external world, but also a means to perform stable computation with a changing nervous system.

## Results

**Figure 1:**
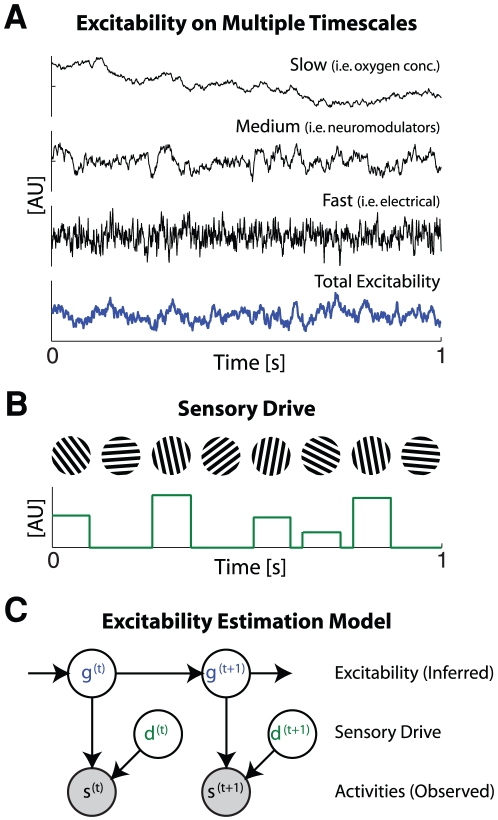
An excitability estimation model. A) Examples of fluctuations in excitability on multiple timescales. We assume that excitability fluctuates due to multiple causes – both slowly fluctuating (e.g. oxygen concentration) and quickly fluctuating (e.g. the activities of neighboring neurons). B) Example of sensory drive. We assume that sensory drive is sparse – non-zero values are relatively rare – and independent from one time-step to the next. C) A schematic depicting the relationship between the excitability, sensory drive, and presynaptic activity. At each time presynaptic activity s is observed. We assume that this activity is the product of the two hidden variables: sensory drive d and excitability (gain) g. Here we assume that the excitability at each time depends on the excitability at the previous time-step. Using this model we can estimate excitability given observations of presynaptic activity, and subsequently normalize postsynaptic responses by this excitability to produce a more stable output.

The central problem in estimating intrinsic fluctuations in excitability is that firing rate information is ambiguous. High firing rates may occur because of strong sensory drive or, alternatively, because presynaptic neurons are highly excitable. In order to adapt in a way that preserves sensory information, the nervous system needs to resolve this ambiguity. Specifically, the nervous system can use information about the way excitability typically changes over time and information about the way sensory drive typically changes over time to estimate presynaptic excitability from presynaptic activity. Here we assume that excitability drifts on multiple timescales around a steady state point [11] and that sensory drive is sparse [12]. The multiple timescales of fluctuations in excitability are meant to mimic the different sources of noise in the nervous system, and we assume that each timescale contributes equally to the total fluctuations in excitability. On short timescales electrical activity and neuromodulators may affect excitability, while on longer timescales excitability may be driven by oxygen concentrations or hormones (Fig 1A). On the longest timescales synaptic pruning, neuronal loss, and aging may all cause changes in excitability. To distinguish between excitability and sensory drive the way that excitability changes over time needs to differ from the way sensory drive changes over time. Here we assume that sensory drive is sparse and changes rapidly. This assumption implies that neurons typically receive low drive and then, only occasionally, receive very high drive (Fig 1B). Here we assume that the total presynaptic activity is the product of this fluctuating excitability and a sparse sensory drive, and that postsynaptic neurons solve the statistical problem of estimating excitability in order to remove it from their output.

Given observations of noisy presynaptic activity, our adaptation model estimates the excitability of the presynaptic neuron on each timescale using approximate Bayesian inference (an assumed density filter). We then model the response of the postsynaptic neuron by the observed presynaptic activity divided by the total estimate of the presynaptic excitability (see Methods for details). The effect of this optimal adaptation rule is to normalize the inputs from each presynaptic neuron. Inputs from presynaptic neurons with high excitability will tend to have low gain, while inputs from neurons with low excitability will tend to have high gain. Under this rule, short term increases in firing rate are typically attributed to high sensory drive while prolonged increases in firing rates are attributed to high excitability. In the following sections we ask how this excitability estimation model of adaptation relates to synaptic properties and to adaptation phenomena measured in primary visual cortex.

**Figure 2:**
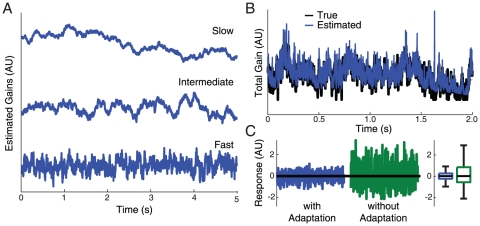
Reducing response variability. A simulated neuron receives input from an orientation tuned neuron whose excitability is fluctuating. A) Estimated pre-synaptic fluctuations on three timescales (slow tau = 5 min, intermediate tau = 500 ms, and fast tau = 50 ms). B) The total pre-synaptic gain in this simulation and the optimal estimate given noisy observations of the pre-synaptic activity. C) The post-synaptic response – presynaptic activity normalized by the estimated gain – to a single orientation with and without adaptation. Boxes denote the inter-quartile range; whiskers denote 1.5 times the inter-quartile range. Outliers have been removed for clarity. By cancelling out fluctuations in pre-synaptic excitability the adaptation model can substantially reduce response variability.

First, using a simulation of a single synapse, we illustrate that estimating presynaptic excitability and normalizing postsynaptic responses by these estimates makes neural output more stable (Fig 2). That is, we show that using an excitability estimation strategy to adapt to presynaptic fluctuations can reduce variability in postsynaptic responses. To give a concrete example, we simulate a presynaptic neuron whose excitability is fluctuating on multiple timescales (Fig 2A). The response of the presynaptic neuron is then the total excitability multiplied by a sparse sensory drive. Given this presynaptic activity, we then use our excitability estimation model to simulate the response of a postsynaptic neuron. With this statistical structure, we can reliably estimate the total presynaptic excitability, 73±1% variance explained (Fig 2B). Importantly, normalizing the response by the estimated fluctuations at each time step gives a much more stable postsynaptic response to the same sensory input than a model without adaptation (Fig 2C). While a certain level of response variability may serve computational purposes [13], [14], reducing response variability using such an adaptation rule could prevent runaway variability that would lead to perceptual errors [15].

**Figure 3:**
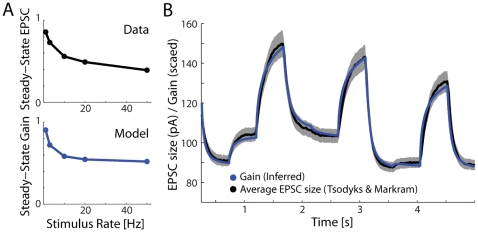
Comparison with short-term synaptic depression data and previous model. A) Low-pass filtering with short-term synaptic depression. Steady-state EPSC size (as a fraction of control) for real data, adapted from [16], (top) and the steady-state gain from the excitability estimation model as the input is varied (bottom). B) A comparison of the EPSC magnitudes predicted by the Tsodyks and Markram model (black +/− SEM, averaged over many spike-train simulations) with the gains predicted by our excitability estimation model (blue). The model proposed by Tsodyks & Markram (1997) allows for a closed-form calculation of successive EPSC magnitudes, given a set of presynaptic spikes. The close match indicates that the changes in average EPSC magnitude are consistent with those that would be produced by a mechanism in service of excitability estimation.

At the level of individual synapses, what properties would be required to approximate optimal adaptation? The adaptation rule we present here, normalizing by estimated presynaptic excitability, is based solely on statistical descriptions of how sensory drive and presynaptic excitability change over time. However, one of the main characteristics of this rule is that synaptic strength increases slowly in the absence of presynaptic activity and decreases quickly in the presence of presynaptic activity. These effects roughly correspond to biophysical descriptions of synaptic depletion and recovery. Indeed experimentally observed properties of short-term synaptic depression [16] are accurately modeled by excitability estimation (Fig 3A). Additionally, a prominent model [17] based on synaptic depletion and recovery and calibrated with electrophysiological results shows a very similar time-course to the one predicted by our excitability estimation model (Fig 3B). Since the biophysical model relies on precise spike timing, these EPSC results shown the average response over many (1000) simulated inhomogeneous Poisson spike trains. This firing rate response can then be compared to the estimated excitability. Each of these results was fit with one free parameter (a scaling factor). The other parameters in the model (the timescales and variability for each timescale) are fixed by the assumption that each timescale contributes equally to the total variance. However, the fact that the model can describe these data raises the possibility that the seemingly counterproductive depletion of synaptic vesicles may serve an important algorithmic purpose – allowing an unstable nervous system to compute reliably.

In addition to neural responses at a single synapse, the excitability estimation model may also be used to describe extracellular responses to the repeated stimuli used in typical physiological experiments. One particularly well studied system for this type of experiment is primary visual cortex. Here, recent experiments have found that orientation-selective neurons, when adapted with a stimulus of one orientation, shift their preferred stimulus orientation away from the adapting stimulus [18], [19], [20]. This kind of response has been described in many neural recordings [18] as well as in human psychophysics [21], [22]. The excitability estimation model naturally reproduces these repulsive tuning curve shifts in a simple model network where each synapse implements the statistically optimal adaptation rule described above.

These physiological adaptation effects follow interesting temporal profiles. If the adapting stimulus lies on one flank of the tuning curve, then the response at this “near flank” is quickly reduced. Only later is an increased response observed on the far flank. Interestingly, this far-flank facilitation often resulted in an increase in the magnitude of the response at the (shifted) peak of the tuning curve. The response profile was not merely translated away from the adapting stimulus, but instead underwent changes which occur on at least two separate time scales [23]. While the overall effect is one of repulsion, the dynamics of the tuning shift displayed additional subtleties and this time-course can constrain potential models.

**Figure 4:**
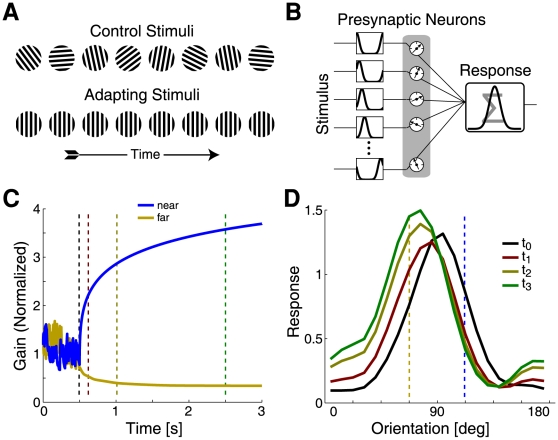
Simulation of repulsive tuning curve adaptation. A) The stimuli presented during the control (upper) and adaptation (lower) epochs of the sensory adaptation simulations; these are meant to replicate the stimuli used in Dragoi et al. B) A schematic of the network model used to simulate orientation adaptation. A population of presynaptic neurons, each with its own orientation tuning curve, reacts to the stimulus. These presynaptic activities are then modulated by the presynaptic excitability, weighted, and summed to produce the postsynaptic response. While the synaptic weights remain constant, the excitability estimates are updated over time according to the model. C) The time course of two sets of gains during a period of adaptation to a single stimulus. The gains correspond to presynaptic inputs whose preferred orientations are on the near (blue) and far (yellow) flanks of the control tuning curve. The dashed lines indicate the time points at which the control tuning curve was measured (black), as well as three successive adapted tuning curves (red, gold, and green). D) The control tuning curve (black), as well as three successive adapted tuning curves corresponding to the time points indicated in A.

Using a feed-forward network of orientation tuned neurons with adapting synapses (Fig 4B), our model explains both the repulsive shift observed in the orientation tuning curve after adaptation, as well as the separate time scales which govern the effects on the two flanks (Fig 4C and D). This separation of time scales results from the assumption of sparse activities. Recall that at the synaptic level, increases in presynaptic activity result in fast decreases of synaptic strength, while decreases in presynaptic activity result in slow increases of synaptic strength. Here, inputs that are tuned to the adapting stimulus (near-flank) suddenly increase their activity. This persistent increase is quickly attributed to an increase in excitability (Fig 4C, blue), and thus the influence of the drive from these synapses decreases quickly. At the far-flank, there is a persistent decrease in activity, which is attributed to a decrease in excitability (Fig 4C, yellow) and results in a slow increase in the influence of these inputs.

**Figure 5:**
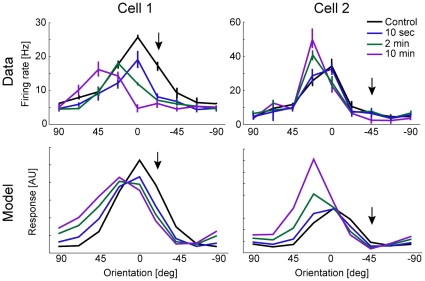
Comparison with tuning curve adaptation data. Control and adapted tuning curves from two real example neurons, adapted from Dragoi et al. (2000) (top). Control and adapted tuning curves from two model neurons whose initial tuning weights were modified to match the electrophysiological data (bottom). In these two cases, the network tuning parameters are slightly different, but the estimation model and parameters are the same.

Interestingly, Dragoi et al. [18] observed strong heterogeneity in the physiological responses of individual neurons to adaptation in cat primary visual cortex. Using the excitability estimation model we can examine how variations in neural properties translate into variability in adaptation responses. We performed two simulations in which the parameters governing the presynaptic excitability were kept constant, but the tuning properties of the adapting neuron were varied (adjusted to produce control tuning curves which approximately matched two real neurons). Aside from the tuning properties of the presynaptic neurons, these simulations used no free parameters. As above, we assume that the contribution of each timescale to presynaptic excitability is the same. However, these simulation results reliably predict the adapted responses of these two neurons (Fig 5). The qualitative differences between the observed tuning curve shifts can be explained by differences in the tuning widths of the presynaptic inputs and differences in the tuning properties of the model neurons. These predictions can be tested in future electrophysiological experiments.

Finally, it is important to ask how robust the assumptions made under the excitability estimation model are. Since a variety of biological factors (oxygen concentration, neuromodulators, etc) appear to affect pre-synaptic excitability, the assumption of multi-timescale fluctuations seems reasonable. Sampling from the generative model, this approach stably transmits sensory drive with 88±0.3% variance explained. Although the assumed density filter used here assumes that sensory drive is sparse and temporally uncorrelated, we can examine how well it performs when the drive does have temporal structure. Using 1/f noise [24] the sensory drive is reconstructed with 36±1% variance explained. Removing the two fastest timescales of excitability fluctuations (2 ms and 5 ms), 1/f noise can be reconstructed with 59±1% variance explained. Removing the three fastest timescales (2, 5, and 18 ms), 1/f noise can be reconstructed with 75±1% variance explained. So while the excitability estimation model presented here is not optimal for temporally correlated sensory drive, structured (1/f) drive can be reconstructed with some accuracy, and accuracy increases as the timescales of fluctuations in excitability and sensory drive are separated.

## Discussion

Here we have shown that several short term and medium term adaptation effects are consistent with a strategy whereby the nervous system attempts to compute reliably in the presence of constantly changing intrinsic excitabilities. Both short-term adaptation phenomena, those occurring over tens or hundreds of milliseconds [17], [25], [26] and medium timescale adaptation phenomena occurring over seconds to minutes [18], [20], have often been explained as synaptic or neural “fatigue”. Under this interpretation, adaptation can be viewed as a failure by the system to achieve the proper response due to the temporary depletion of resources. The adaptation rule that we have presented here describes how such phenomena may be the result postsynaptic neurons solving the statistical problem of estimating presynaptic excitability and canceling fluctuations in excitability. By assuming that excitability drifts on many timescales and that sensory drive is sparse, this model describes both short-term adaptation at individual synapses as well as changes in orientation tuning during typical medium timescale adaptation experiments.

There is a long history of normalization models in psychophysics. These models [27] suggest that visual adaptation results from two effects: error-correction mechanisms [28] and dynamic range optimization [29]. By attributing persistent activity to fluctuations in excitability, the model presented here provides an instantiation of these models and performs both error-correction and dynamic range optimization (gain control, see Supplementary Note S2 and Fig S2 for details). However, under classical proportional gain adjustment schemes [30], there is a single timescale of adaptation and adaptation is symmetric. That is, adaptation to an error in one direction occurs just as quickly as adaptation to an error in the opposite direction. Here, by assuming that excitability fluctuates on multiple timescales and that sensory drive is sparse, adaptation is both multi-timescale and asymmetric. Both of these factors appear to be important in explaining the time-course and structure of synaptic depression and repulsive tuning curve adaptation in V1.

More recently, generalizations of normalization-type models have proposed that the nervous system adapts to optimize the amount of information transmitted by a sensory system which is limited by noise or the availability of neural resources [10], [31]. The principle behind these approaches (information maximization at the perceptual level) is different from the one presented here (stability at the synaptic level). At first glance, models in which the entire nervous system adapts to the changing statistics of the natural world are in conflict with the model presented here where individual synapses attempt to preserve local stability. However, it is important to note that these two principles are not mutually exclusive. Although some evidence suggests that information maximization may not explain adaptation across multiple cortical levels [32], the brain is likely to implement strategies that are useful for multiple purposes, and a range of models address the mechanisms by which the nervous system may implement perceptual adaptation [23], [33], [34].

However, several recent papers have considered information maximization at the cellular or synaptic level [11], [35], [36]. Notably, Pfister et al. [37] consider synapses inferring presynaptic membrane potential given only spike observations. As with the excitability estimation model, this framework accurately describes several characteristics of short-term synaptic depression and provides a normative alternative to more descriptive, biophysical models. In many ways, the excitability estimation model presented here can be interpreted as a system which attempts to efficiently code sensory drive across synapses, even in the presence of multi-timescale intrinsic noise.

A number of computational roles for synaptic depression have been suggested such as decorrelating inputs [38] or adjusting a neuron's dynamic range (gain-control) [39]. For neurons to encode signals from both slowly and rapidly firing presynaptic inputs there must be some means for neurons to lower the gain of rapidly firing afferents while increasing the gain of slowly firing afferents. Without this type of gain-control, any modulation of the low-activity inputs will be masked by noise from the inputs with high activity. In the model we present here ongoing activity is always attributed to presynaptic activity so that changes in background activity are naturally normalized by the excitability.

Synaptic depression has also been considered as a mechanism for a number of cellular-level phenomena including direction selectivity and contrast adaptation [40], cross-orientation suppression [41], and spatial-phase adaptation [42]. To our knowledge repulsive shifts in orientation tuning have not yet been explained by synaptic depression alone. However, it seems likely that such repulsive shifts could be a result of a type of short-term synaptic depression. Rather than studying typical models of synaptic depression as a specific phenomenon, here we treat synaptic adaptation in general, as the result of excitability estimation. That such a framework can also explain cellular adaptation on longer timescales suggests that a normative principle based on stable computation may be a useful way to link synaptic properties with cellular response properties.

In explaining medium timescale adaptation phenomena we have focused on “repulsive” adaptation, where tuning curves are shifted away from an adapting stimulus. At the perceptual level, examples of repulsive adaptation include the tilt after-effect [18] and the motion after-effect [43], [44]. In our model, repulsive tuning curve changes are understood as rational errors committed by a sensory system which assumes sparseness about neural drive. The optimal adaptation rule derives from our assumptions about the statistics of typical neural drive and typical changes in excitability. In an area of the brain where stimuli typically change slowly relative to neural excitability adaptation would have the opposite sign and responses to repeated stimuli should be stronger. This may well be the case for the motion selective area MT where adaptation of the opposite sign, attractive adaptation, is observed [45]. The estimation problem solved by neurons may thus involve estimation of both intrinsic excitabilities and extrinsic drive variables.

In the model presented here, the assumption of sparse, temporally uncorrelated sensory drive is unlikely to reflect the true statistics of an external variable. Natural stimuli are spatially and temporally correlated on a range of scales [46], and there is substantial evidence that temporal structure in extrinsic drive variables may be important for both neural coding and learning [47], [48], [49], [50]. Being able to disambiguate fluctuations in excitability from sensory drive requires some distinction between their temporal structures. While assuming sensory drive is sparse and temporally uncorrelated makes disambiguation easier, it may be more likely that sensory drive is temporally correlated (as 1/f, for instance). Although correlated sensory drive may be more difficult to distinguish from fluctuations in excitability, it is important to note that the credit-assignment problem that we model here operates on a processed version of sensory signals. These processed sensory signals may be substantially sparser and less temporally correlated than the features of natural images [51], [52].

More generally, the model described here uses a simple linear response model and thus clearly only implements a rough approximation to the problem solved by the nervous system. For example, the inputs in the model are independent of one another, and the excitabilities of each presynaptic neuron are estimated and adapted separately. This framing ignores the correlations which are known to exist in the firing patterns of neural populations, and such correlations may be crucial in explaining other aspects of physiological adaptation phenomena. In addition, the simple network model that we use to simulate orientation tuning omits many of the properties which such thalamo-cortical networks are known to exhibit. We chose the simple model here as it allows us to compactly solve the statistical problem of estimating excitability. However, stable computation is an appealing general principle.

In a statistically optimal system adaptation is determined by assumptions about the way both the nervous system and sensory drive change over time. When these assumptions are violated, for example by experiments that repeatedly present stimuli that are rare in the natural environment, phenomena such as the tilt and motion after-effects are the result. Underlying our analysis of adaptation is the assumption that the stimuli which lead to effects such as the tilt aftereffect and the motion aftereffect are in fact very rare in the natural environment – and the price the nervous system pays for adaptation under normal situations is thus very small. These unusual stimuli fool the perceptual system by mimicking a situation in which the excitability of some neurons has been increased (and others possible decreased). We propose that physiological and perceptual sensory adaptation stems from this fundamental ambiguity that exists between the intensity of sensory stimuli and the excitability of the neurons that process these signals.

## Methods

### An Excitability Estimation Model of Adaptation

#### Generative Model

We assume that a post-synaptic neuron seeks to estimate fluctuations in its inputs that occur over time scales ranging from a few milliseconds to minutes. To accomplish this goal, the neuron must estimate the excitability fluctuations of its inputs at each of these time scales. We refer to these estimates of excitability as gains.

Throughout the following, we use *M* = 10 timescales 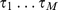, linearly spaced on a log scale from a few milliseconds (for 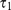 = 2ms), up to several minutes (for 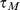 = 5.5min). The temporal dynamics of the *M* gains are assumed to be independent random walks with zero-mean drift (Ornstein–Uhlenbeck processes), with a variance from one time step to the next that is inversely proportional to the time scale:
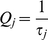
Each gain decays towards zero at a rate 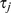, and setting the process variance 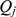 inversely proportional to this rate ensures that the total (stationary) variance associated with each of the time scales is the same. The evolution of the gains is thus specified by the linear dynamical system
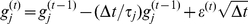
Where 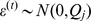 describes the process noise, independent for each individual gain. The overall gain 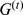, the total excitability of the presynaptic neuron, is determined by the sum of the individual gains plus 1:
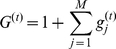
We further assume that synaptic drives are sparsely distributed under natural stimulus conditions [52], meaning that high drive values are much less common than low ones. We formalize this assumption by placing an exponential prior distribution on the drives, i.e., 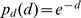. Lastly, we assume that the presynaptic activity at each time step is the product of this sparsely-distributed true drive and the total current gain estimate. This gives the observation model
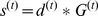
For the simulations presented here 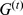 is close to 1 and *s* is positive. To estimate presynaptic excitability we then need to be able to update a probability distribution over the vector 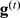 given the a new observation 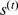. Given the exponential prior distribution over 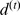, and the assumption that 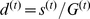, we can employ a variable transformation and marginalize over 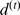 to find the following likelihood distribution for 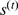:
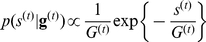
This exponential likelihood distribution incorporates our sparseness assumption, and indicates that high input values are much less common, and therefore more informative, than low input values. This asymmetry will influence the dynamics of the gain estimates by allowing gain increases to be detected more quickly and with less uncertainty than gain decreases.

#### Estimating Excitability

We have described a statistical model whose conditional independence structure is equivalent to that of the state space model, a standard framework for describing the dynamics of normally distributed variables. However, the likelihood distribution for *s* that we derive from our sparseness assumption is non-Gaussian. To perform approximate Bayesian inference with this likelihood, we use assumed density filtering (ADF) [53]. Briefly, we replace the true posterior 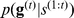 at each time-step with a Gaussian (Laplace approximation), which will allow us to estimate the synaptic gains in response to any series of stimulus values (see Supplementary Note S1 for details). In practice, we find this approximation to be quite stable (Fig S1).

Given the assumptions about how 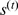 is generated this assumed density filter allows us to maintain a probability distribution over 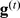 and, thus, an estimate of the total presynaptic excitability 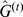. The goal of this model is then to remove the fluctuating excitability from the postsynaptic response. In modeling a single synapse, we assume that the postsynaptic response is the presynaptic activity normalized by the constantly updated estimate of the total presynaptic excitability,
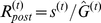



### Short-Term Synaptic Plasticity

#### Reducing Response Variability

To illustrate the role of adaptation in stable computation, we simulate from the generative model described above where both fluctuations in presynaptic excitability and the sensory drive are known (Fig 2). In this case, we simulate a single orientation tuned presynaptic neuron (circular Gaussian tuning curve) driving a post-synaptic neuron with no other inputs. We look at the response of the postsynaptic neuron for randomly presented gratings. Stimulus orientations were drawn uniformly and independently, presented for 50 ms each – this specifies the sensory drive. In this case we explicitly simulate fluctuations in presynaptic excitability using the same multi-timescale linear dynamical system described above (see Fig 2A and B). We model the response of an adapting postsynaptic neuron then by 
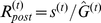
 where 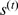 is the noisy input from the presynaptic neuron and 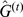 is the total estimated presynaptic gain, as described above (see Fig 2B). We assume that the response of a non-adapting postsynaptic neuron is simply proportional to the input 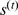 and a gain of 1 is assumed. The parameters for the timescales and their associated variability are all fixed as above (i.e. 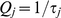).

#### Modeling Short-term Synaptic Depression

We then use this model of gain dynamics to model the short-term changes in strength of a single synapse. We compare the resulting gain changes to a model based on neurotransmitter depletion [17]. Under the biophysical model synaptic resources are described as being effective, inactive, or recovered, and the fraction of resources in each state evolves according to kinetics that depend on the times of recent presynaptic spikes. The postsynaptic current at a given time is proportional to the fraction of resources in the effective state. Assuming that the time between spikes is much longer than the timescale of inactivation (typically ∼3ms) the size of excitatory postsynaptic currents (EPSC) follows
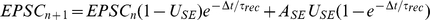
where 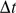 denotes the time interval between the *n*th and *(n+1)*th spike, 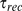 denotes the timescale of recovery, 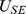 denotes the utilization parameter, and 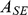 denotes the absolute synaptic efficacy. For our simulation we use the same parameters as [17]: 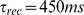, 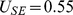, 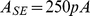, and the input level (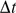) was drawn randomly every 500ms from a uniform distribution. Since this biophysical model operates on the precise spike timings, we drew 1000 inhomogeneous Poisson spike trains from the same underlying firing rate. Fig 3B shows the average, firing rate response. For our model, we use the same sensory drive (the input level 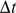) and kept the true presynaptic excitability fixed during the simulation. Given this presynaptic activity, we then estimate the total presynaptic excitability 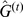 using the methods described in the first sections. In this case the estimated presynaptic excitability is very different from the true presynaptic excitability, which is fixed. Instead, presynaptic activity which is unlikely to be generated by sensory drive, given our assumptions, is attributed to fluctuations in presynaptic excitability.

To fit the data from [16] we use the same techniques. We assume that the sensory drive is equivalent to the stimulus frequency and that the true presynaptic excitability is fixed. Given this presynaptic activity (the product of the sensory drive and generative excitability) we then estimate the presynaptic excitability 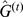.

To model results for [16] as well as [17] we fit a single scaling parameter to each. Importantly, this is the only free parameter. The other parameters (the timescales 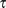 and their associated variabilities 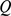) are fixed by the assumption that each timescale contributes equally to the total variance.

### Orientation Tuning Adaptation

#### Network Model

We also apply the model of gain dynamics to a simple network model of orientation tuning in primary visual cortex. In this model, a single postsynaptic cell receives input from *N* presynaptic cells (N = 10 for the results presented here). Each of the *N* synapses has a full complement of *M* gains, and the gains at each synapse *i* are estimated independently in response to the presynaptic activity observed at each time step, 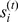. The activity level of the postsynaptic cell at time *t* is determined by these excitability-normalized inputs:
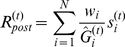
Here, 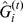 is the total estimated excitability at time *t* for *i*th presynaptic input. Note that the postsynaptic cell is completely linear, as its response computed as a weighted sum of its inputs at each time step. The *w_i_*'s are the fixed components of the synaptic strengths, and follow a circular Gaussian tuning curve profile:
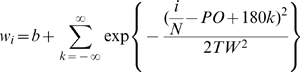
The presynaptic cells are also assumed to have circular Gaussian tuning curves, with preferred orientations evenly spaced every 180/*N* degrees. Given this tuning we can then generate the response 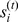 of each presynaptic neuron at each time. In this simple model, the presynaptic cells do not have their own input gains and do not undergo adaptation.

At each time step, the stimulus causes each of the presynaptic cells to become active according to its tuning curve. This stimulus drive is in addition to a lower, spontaneous level of activity *b* which is always present on all of the presynaptic neurons. This input profile is a simple approximation of the orientation-tuned inputs which are known to feed neurons in primary visual cortex. This simple model omits prominent features of real orientation-selectivity networks, including nonlinear responses and recurrent connections, but it is able to reproduce several important adaptation phenomena (Figs 4 and 5).

We should note that, in addition to the pre-adaptation tuning curve, the tuning widths of the presynaptic neurons affect the specific time-course and shape of adapted tuning curves. The input tuning widths for the two simulations in Figure 5 were randomly perturbed. Importantly, this network model and the results presented here use no free parameters aside from those which determine the initial tuning curves. The timescales and associated variability on each synapse were fixed according to the single-synapse model described above. The synaptic strengths were fit using maximum likelihood (Gaussian noise model) to match the initial tuning curves for the two example cells (Fig 5), and the preferred orientations of the presynaptic cells were evenly spaced.

#### Stimuli

Each orientation adaptation simulation is divided into two epochs (Fig 4A). In the first “control” epoch, the stimulus orientations are drawn uniformly and independently and presented for one second each (i.e., white noise; see Fig 4A, upper). In the second “adaptation” epoch, a single stimulus orientation is chosen and is presented continuously for the duration of the simulation (see Fig 4A, lower). During this adaptation period, the presynaptic cell whose preferred orientation is closest to the adaptation stimulus is maximally active, and all of the other inputs are active to a lesser degree. The exact degree of activation is determined by the circular Gaussian tuning curve associated with each presynaptic cell. A schematic of the complete model is shown in Figure 4B.

Again, as with the simulations of single synapses we assume that the true presynaptic excitability is fixed. In this case the estimated presynaptic excitability of each synapse is very different from the true presynaptic excitability. Instead, presynaptic activity which is unlikely to be generated by sensory drive, given our assumptions, is attributed to fluctuations in presynaptic excitability.

## Supporting Information

Figure S1 The assumed density approximation in 2D. Calculating the true posterior at each time-step is difficult to do analytically, since the likelihood is non-Gaussian (left column). However, we can approximate the posterior with a Gaussian at each time-step (right column, ADF). Here we show a single time-step of this approximation for 2-dimensions, where s = 2, cov_t = 2.5I, and g(t-1) = [0.5; 0.5] (top row) or g(t-1) = [1.5; 1.5] (bottom row). The blue line denotes the maxima of the likelihood, the blue circle denotes the maximum of the prior, and the black cross denotes the maximum of the posterior.
(0.74 MB EPS)

Figure S2 Gain control. The excitability estimation model naturally reproduces many aspects of gain control. Input rates are naturally normalized by ongoing activity. In this simulation a neuron receives two synaptic inputs with average rates of 100Hz and 10Hz. (left) 50% modulation of the 100Hz input, produces ∼12% modulation in the output with adaptation and ∼50% modulation without adaptation. (middle) 50% modulation of the 10Hz input, produces ∼15% modulation in the output with adaptation and 5% without modulation. (right) 5% modulation of the 100Hz input produces <5% modulation in the output with adaptation. Blue and green denote the rates of the 100Hz and 10Hz input respectively.
(3.12 MB EPS)

Note S1 Assumed density filtering.
(0.04 MB DOC)

Note S2 Excitability estimation and gain control.
(0.03 MB DOC)
