# Supplementary material for: Sensory Adaptation and Short Term Plasticity as Bayesian Correction for a Changing Brain
Source: PLoS One. 2010 Aug 26;5(8):e12436. doi: 10.1371/journal.pone.0012436 (PMC2928744; doi:10.1371/journal.pone.0012436)
Supplement: Note S1 — Assumed density filtering. (0.04 MB DOC) [file pone.0012436.s003.doc]

***Supplementary Note S1: Assumed Density Filtering***

In order to estimate the gains at each time-step, we need to compute the posterior

$$p(\mathbf{g}_{t}|s_{1:t})\propto p(s_{t}|\mathbf{g}_{t})p(\mathbf{g}_{t}|s_{1:t-1})$$

Since the likelihood is non-Gaussian, this is difficult to do analytically. However, we can use the standard assumed density filtering (ADF) approach to approximate the posterior with a Gaussian at each time-step [1]. Using the likelihood and the fact that the prior is Gaussian, the log-posterior is given by

$$\log(p(\mathbf{g}_{t}|s_{1:t})=-\log G_{t}-\frac{s_{t}}{G_{t}}-\frac{1}{2}(\mathbf{g}_{t}-A\mathbf{g}_{t-1})^{T}\Sigma_{t}^{-1}(\mathbf{g}_{t}-A\mathbf{g}_{t-1})-\log Z$$

where$\Sigma_{t}=A\Sigma_{t-1}A^{T}+Q$, A denotes the transition matrix, Q denotes the process noise covariance, and Z denotes the partition function, which is constant in g. We then use a Laplace approximation [2], replacing the posterior with a Gaussian whose mean is the posterior mode and whose covariance matrix is the negative inverse of the Hessian of the log-posterior (evaluated at the mode). We find the mode using a conjugate gradient, approximate line-search method (http://www.kyb.tuebingen.mpg.de/bs/people/carl/code/minimize/), where the gradient is given by

$$\frac{d\log\left(p(\mathbf{g}|s)\right)}{d\mathbf{g}}=\left[\frac{-1}{G}+\frac{s}{G^{2}}\right]-\Sigma^{-1}(\mathbf{g}-A\mathbf{g}{}_{_{t-1}})$$

and the Hessian is

$$\frac{d^{2}\log\left(p(\mathbf{g}|s)\right)}{d\mathbf{g}d\mathbf{g}^{T}}=\left[\frac{1}{G^{2}}-\frac{2s}{G^{3}}\right]-\Sigma^{-1}$$

## This mean and covariance can then be used iteratively to generate the prior at the next time-step and to obtain a new estimate of g, given a new observation s. It is important to note that, although the likelihood does not have a unique maximum (there are an infinite number of maxima wherever $G=\sum g=s$), the log-likelihood is strictly decreasing away from this hyper-plane. Since the prior (a Gaussian) is strictly log-concave, the posterior is strictly log-concave for $s>0,g>0$, and there is a single global maximum for the true posterior. In practice we find the ADF approximation to be quite stable, and the additional computational costs of finding the posterior-mode at each time-step are negligible (Figure S1).

1. Minka T (2001) Expectation Propagation for Approximate Bayesian Inference. Proceedings of the 17th Conference in Uncertainty in Artificial Intelligence: 362.

2. MacKay DJC (2003) Information Theory, Inference, and Learning Algorithms: Cambridge University Press, 2003.
