# Supplementary material for: Sensory Adaptation and Short Term Plasticity as Bayesian Correction for a Changing Brain
Source: PLoS One. 2010 Aug 26;5(8):e12436. doi: 10.1371/journal.pone.0012436 (PMC2928744; doi:10.1371/journal.pone.0012436)
Supplement: Note S2 — Excitability estimation and gain control. (0.03 MB DOC) [file pone.0012436.s004.doc]

## Supplementary Note S2: Excitability estimation and gain control

In describing synaptic depression, the model we present also captures several important computational features of synapses. Notably, the excitability estimation model yields a type of gain control as a direct consequence of the assumption of sparse input. On-going activity is attributed to the excitability of the presynaptic neuron, and changes in presynaptic activity are automatically scaled by these gains.

To illustrate this point we performed two simulations analogous to those from [1]. In the first simulation a neuron receives two inputs with baseline rates of 10Hz and 100Hz respectively. Successful gain control produces something akin to a Weber-Fechner law where the relative modulation on each input is important rather than the absolute modulation. Similar to the synaptic depression model of [1], we find that a postsynaptic neuron using the excitability estimation rule is sensitive to relative modulation rather than the absolute modulation (Figure S2A). There is non-linearity in the output, as some of the modulation in the inputs is attributed to gain changes. When the modulation is faster than the timescale of the gains it is attributed solely to the drive and this nonlinearity disappears. See Fig 2 in [1] for a comparison.

One potential difficulty with gain control is how the postsynaptic neuron remains tuned. If low presynaptic activity always corresponded to high gain tuning would be lost. Since the excitability estimation model is explicitly attempting to distinguish presynaptic excitability and sensory drive, this is less of a concern. However, as in [1] we find that although tuning broadens it is not eliminated by adaptation. Figure S2B qualitatively reproduces Fig 3 from [1].

The first simulation uses the same parameters as [1]. The second simulation was performed with 21 evenly spaced Gaussian-tuned presynaptic neurons (σ=1), with synaptic weights normally distributed with zero mean and a standard deviation of 1 following [1]. Stimuli were uniformly distributed on [-5,5] and randomly presented at 20Hz.

1. Abbott LF, Varela JA, Sen K, Nelson SB (1997) Synaptic depression and cortical gain control. Science 275: 221.
